# P-1385. Real World Side Effect Profile of Bedaquiline: An analysis of pharmacovigilance data

**DOI:** 10.1093/ofid/ofaf695.1572

**Published:** 2026-01-11

**Authors:** Hassaan Ali Ahmad

**Affiliations:** Faisalabad Medical University, Faisalabad, Punjab, Pakistan

## Abstract

**Background:**

Bedaquiline, approved in 2012 in the United States as the first new drug for multidrug-resistant tuberculosis in over 40 years, showed increased mortality in clinical trials compared to placebo in addition to other side effects. We analyzed real-world side effects reported to the FDA Adverse Event Reporting System (FAERS) to further assess its safety profile.

**Methods:**

The FDA Adverse Event Reporting System (FAERS) database was searched for all adverse events reported for bedaquiline from inception through April 30, 2025. All adverse events reported for bedaquiline were analyzed.

**Results:**

A total of 3,499 adverse event (AE) cases related to Bedaquiline were reported. The majority (84%) of the cases were submitted from 2018 to 2024. Of these, 225 (6.4%) occurred in the United States, while 3,274 (93.6%) were reported from other countries. Among cases with available age data, 86% were between 18–64 years, and 9% were aged 65–85 years. Gender distribution showed 38% female and 46% male, with gender unspecified in 15% of cases. Reports were submitted predominantly by healthcare professionals (98%), with 1.5% submitted by consumers. A total of 3,414 cases (97.6%) were classified as serious adverse events. Off-label use was documented in 11.66% of cases. Death was the reported in 1,095 cases (31.3%). The most frequently reported adverse events were QTc prolongation (15.9%), anemia (9.9%), vomiting (8.0%), peripheral neuropathy (7.0%), hepatotoxicity (6.8%), nausea (6.5%), death (6.2%), dyspnea (5.0%), diarrhea (3.2%), elevated aspartate aminotransferase (3.0%), and hypokalemia (3.0%).

**Conclusion:**

Bedaquiline is associated with a high proportion of serious and potentially fatal adverse events, including QTc prolongation and hepatotoxicity. Continued pharmacovigilance is essential to ensure its safe use, particularly in off-label settings.

**Disclosures:**

All Authors: No reported disclosures

